# Exploring the multidimensional nature of repetitive and restricted behaviors and interests (RRBI) in autism: neuroanatomical correlates and clinical implications

**DOI:** 10.1186/s13229-023-00576-z

**Published:** 2023-11-27

**Authors:** Aline Lefebvre, Nicolas Traut, Amandine Pedoux, Anna Maruani, Anita Beggiato, Monique Elmaleh, David Germanaud, Anouck Amestoy, Myriam Ly-Le Moal, Christopher Chatham, Lorraine Murtagh, Manuel Bouvard, Marianne Alisson, Marion Leboyer, Thomas Bourgeron, Roberto Toro, Guillaume Dumas, Clara Moreau, Richard Delorme

**Affiliations:** 1 Fondation Vallée, GHT Paris Sud, Hospital of Child and Adolescent Psychiatry, Gentilly, France; 2 UMR 3571 CNRS, Human Genetics and Cognitive Functions, Institut Pasteur, Paris, France; 3 Unité de Neuroanatomie Appliquée et Théorique, Institut Pasteur, Paris, France; 4 Department of Child and Adolescent Psychiatry, Robert Debré Hospital, APHP, Paris, France; 5 Department of Pediatric Radiology, Robert-Debré Hospital, APHP, Paris, France; 6 UNIACT Neurospin – INSERM UMR 1129, CEA, Saclay, France; 7 Department of Clinical Genetics, Robert Debré Hospital, APHP, Paris, France; 8 Center for Research and Interdisciplinarity (CRI), Université Paris Cité, Paris, France; 9 Autism Expert Center, Charles Perrens Hospital, Bordeaux, France; 10 Fondation FondaMental, French National Science Foundation, Créteil, France; 11 Institut Roche, Boulogne-Billancourt, France; 12 Roche Pharma Research and Early Development, Neuroscience and Rare Diseases, Roche Innovation Center Basel, F. Hoffmann–La Roche Ltd., Basel, Switzerland; 13 Institut National de la Santé et de la Recherche Médicale (INSERM), U955, Institut Mondor de Recherche Biomédicale, Psychiatrie Translationnelle, Créteil, France; 14 Department of Adult Psychiatry, Henri Mondor and Albert Chenevier Hospital, Créteil, France; 15 Université Paris Cité, Paris, France; 16 Department of Psychiatry, Université de Montreal, CHU Ste Justine Hospital, Montreal, QC Canada; 17 Imaging Genetics Center, Stevens Neuroimaging and Informatics Institute, Keck School of Medicine of USC, Los Angeles, CA USA; 18 Faculty of Medicine, Université Paris-Saclay, Le Kremlin-Bicêtre, France

**Keywords:** RRB, Phenotype, Cortico-striatal-thalamo-cortical loop

## Abstract

**Background:**

Repetitive and restricted behaviors and interests (RRBI) are core symptoms of autism with a complex entity and are commonly categorized into ‘motor-driven’ and ‘cognitively driven’. RRBI symptomatology depends on the individual’s clinical environment limiting the understanding of RRBI physiology, particularly their associated neuroanatomical structures. The complex RRBI heterogeneity needs to explore the whole RRBI spectrum by integrating the clinical context [autistic individuals, their relatives and typical developing (TD) individuals]. We hypothesized that different RRBI dimensions would emerge by exploring the whole spectrum of RRBI and that these dimensions are associated with neuroanatomical signatures—involving cortical and subcortical areas.

**Method:**

A sample of 792 individuals composed of 267 autistic subjects, their 370 first-degree relatives and 155 TD individuals was enrolled in the study. We assessed the whole patterns of RRBI in each individual by using the Repetitive Behavior Scale-Revised and the Yale-Brown Obsessive Compulsive Scale. We estimated brain volumes using MRI scanner for a subsample of the subjects (*n *= 152, 42 ASD, 89 relatives and 13 TD). We first investigated the dimensionality of RRBI by performing a principal component analysis on all items of these scales and included all the sampling population. We then explored the relationship between RRBI-derived factors with brain volumes using linear regression models.

**Results:**

We identified 3 main factors (with 30.3% of the RRBI cumulative variance): Factor 1 (FA1, 12.7%) reflected mainly the ‘motor-driven’ RRBI symptoms; Factor 2 and 3 (respectively, 8.8% and 7.9%) gathered mainly Y-BOCS related items and represented the ‘cognitively driven’ RRBI symptoms. These three factors were significantly associated with the right/left putamen volumes but with opposite effects: FA1 was negatively associated with an increased volume of the right/left putamen conversely to FA2 and FA3 (all uncorrected *p *< 0.05). FA1 was negatively associated with the left amygdala (uncorrected *p *< 0.05), and FA2 was positively associated with the left parietal structure (uncorrected *p *= 0.001).

**Conclusion:**

Our results suggested 3 coherent RRBI dimensions involving the putamen commonly and other structures according to the RRBI dimension. The exploration of the putamen’s integrative role in RSBI needs to be strengthened in further studies.

**Supplementary Information:**

The online version contains supplementary material available at 10.1186/s13229-023-00576-z.

## Introduction

Autism Spectrum Disorders (ASD) are complex conditions characterized by atypical social communication, as well as restricted or stereotyped behaviors and interests (RRBI) (DSM-5, APA), affecting 1–2% of individuals from the general population [69]. One source of this complexity relies on the heterogeneity of symptoms depicted by autistic individuals, specifically when considering the RRBI. These symptoms could be dichotomized in ‘motor-driven’ symptoms gathering the stereotyped movements (e.g. head banging) and the repetitive behaviors, and in ‘cognitively driven’ symptoms, including obsessive–compulsive like symptoms and cognitive inflexibility [39, 56]. The determinants of this differential expression remain largely unknown, although the individual's cognitive skills seem to be a major modulator of RRBI. Autistic individuals with a comorbid intellectual deficiency predominantly display motor-related symptoms, cognitively driven symptoms, if they exist, are probably underestimated due to a reporting bias. In contrast, autistic individuals without such deficiency express more those which are cognitively driven [15]. Other modulators affect the expressiveness of these symptoms, such as the individual's age [18] or the associated social communication skills themselves. Studies exploring the familial aggregation of RRBI in autism report that the repetitive/stereotyped behaviors are mainly observed in probands and the obsessive-like symptoms in their non-affected relatives, independently of their cognitive abilities [29, 67]. The apparent dichotomy of the RRBI is also reinforced by the use of distinct screening tools, opposing those dedicated to the exploration of RRBI in the context of autism, such as the Repetitive Behaviors Scale-Revised (RBS-R, [35]) and those measuring them in individuals with obsessive–compulsive disorders with the Y-BOCS as a gold standard [23]. However, contrary to an approach divided into repetitive behaviors for some individuals and obsessive symptoms for others, some authors have developed new tools to consider the wide diversity of RRBI better and offer a unifying approach to these symptoms. Although preliminary and performed in the general population, one study showed a high intra-familial correlation of RRBI, whether the index case has associated autistic symptoms [18]. These findings encourage researchers to reconsider the nosology of RRBI and to adopt a more dimensional approach than a categorical to these symptoms.

It also seems consistent with what the literature reports about the involvement of a similar pattern of brain structures in RRBI. Animal models suggest a critical role of the cortico-basal-ganglia-thalamo-cortical loop in the emergence and maintenance of RRBI [12]. Induced deletion of Shank3 (*SHANK3* being a major gene associated with autism) in inhibitory neurons of the striatum results in repetitive/stereotyped-like behaviors, and conversely, deletion of Shank3 in excitatory neurons of the prefrontal cortex results in excessive self-grooming behaviors considered as an equivalent of obsessive–compulsive symptoms [4]. In humans, studies exploring RRBI in autistic individuals also report abnormalities in subcortical structures, specifically the striatum. For example, using T1-weighted anatomical images from the Autism Brain Imaging Data Exchange, Schuetze et al. [58] reported that stereotyped behaviors were positively associated with increased bilateral globus pallidus surface area. Paralleling these findings, the ENIGMA-OCD working group observed larger thalamic volume affecting the lateral, pulvinar, and ventral regions in children with OCD [66]. However, another mega-analysis by the OCD Brain Imaging Consortium (OBIC) pointed more toward the ventrolateral and dorsomedial prefrontal in obsessive–compulsive behaviors in humans [20]. Moreover, multiple strands of evidence indicate deviations in brain growth and maturation trajectories rather than static alterations in autism and OCD [25, 41, 49].

Altogether the literature presents a certain coherence between the results of clinical, genetic and brain imaging studies suggesting a commonality of the RRBI with a differential expression according to the individual phenotypic characteristics. To better understand the heterogeneity and familial patterns of RRBI in autism, as well as the structural brain abnormalities that underlie them, we performed this study exploring RRBI in a sample of 792 individuals gathering autistic patients (*n *= 267), their non-affected first-degree relatives (*n *= 370) and typically developing individuals from the general population (TD) (*n *= 155). To embrace the diversity of the whole pattern of RRBI, we explored each enrolled individual with the Repetitive Behavior Scale-Revised (RBS-R) [35] and the Yale-Brown Obsessive Compulsive Scale (Y-BOCS) [23], both considered as gold standard questionnaires to explore repetitive/stereotyped behaviors and obsessive–compulsive symptoms in clinical populations. We then ran a factor analysis on all RRBI to apprehend further the distribution of these symptoms independently of the subject's status (affected, non-affected relatives, or typically developing participants). We finally performed a multiple linear regression model on a subsample of 152 subjects to explore the relationship between these RRBI-related factors, and the cortical/subcortical brain volumes based on MRI. We hypothesized that two main factors would emerge from the factor analysis, mirroring the dichotomized model of RRBI (repetitive/stereotyped *vs* obsessive/compulsive symptoms), but displaying common and specific associations with brain structures of the cortico-striatal-thalamo-cortical loop.

## Methods

### Ethics

The study was granted approval by the local Ethics Committee (ref: 2008-A00019-46) and registered in a public trial registry (NCT02628808). The study was carried out in accordance with Good Clinical Practice (ICH GCP) standards. Written informed consent was obtained from all participants. For patients who were unable to consent for themselves, a parent or legal guardian consented to the study on their behalf.

### Participants

A sample of 792 individuals composed of 267 autistic subjects; their 370 first-degree relatives and 155 individuals from the general population with typical development (TD) were enrolled in the study at the Child and Adolescent Psychiatry Department, Robert Debre Hospital, Paris (France). Their demographic and clinical characteristics are reported in Table 1. Participants' clinical assessment procedures followed previously described methods [39]. ASD diagnosis was based on DSM-IV-TR/5 criteria and made by summing the information from the Autism Diagnosis Interview-Revised (ADI-R), the Autism Diagnostic Observation Scale—second edition (ADOS-2), and clinical reports from experts in the field, who made the final diagnostic decision. The non-verbal cognitive abilities were assessed using the Wechsler Intelligence Scales adapted to age or the Raven’s Progressive Matrices (RPM) for those with poor (or lack) verbal abilities [52]. Those with low, mild, or below intellectual disabilities were excluded (IQ < 70).

**Table 1:** Clinical and demographic characteristics of the individuals enrolled in the study

	ASD (*N *= 267)	Relatives (*N *= 370)	Controls (*N *= 155)	*R*^2^	*F*	*p* value
Age	17.7 (12.9)	38.0 (17.2)	38.0 (14.9)	0.3	139.4	< 0.001
Sex—male ratio (%, n)	86%, 230	57%, 174	55%, 86	113.4*	–	< 0.001
OCD (%, *n*)	11.61%, 31	3.24%, 12	0.65%, 1	0.09	–	< 0.001
AD (%, *n*)	14.23%, 38	10%, 37	1.94%, 3	0.06	–	< 0.001
TD (%, *n*)	10.49%, 28	5.95%, 22	4.52%, 7	0.06	–	< 0.001
TS (%, *n*)	1.49%, 4	0.27%, 1	–	0.08	–	0.15
NVIQ	95.6 (23.2)	113.3 (14.7)	100.2 (19.8)	0.1	32.0	< 0.001
SRS T-score	72.2 (12.6)	46.0 (8.5)	44.4 (7.8)	0.6	556.6	< 0.001
ADI—A	16.4 (9.5)	–	–	–	–	–
ADI—B	11.5 (7.8)	–	–	–	–	–
ADI—C	4.9 (3.5)	–	–	–	–	–
ADI—D	2.6 (1.7)	–	–	–	–	–
ADOS-CSS	4.6 (2.1)	–	–	–	–	–

*ASD* Autistic individuals, *OCD* Obsessive–Compulsive Disorder, *AD* Anxiety Disorders, *TD* Tic Disorders, *TS* Tourette Syndrome, *NVIQ* Non-verbal IQ, *ADI-A* ADI-Social interaction domain score, *ADI-B* ADI-Communication domain score, *ADI-C* Stereotypes and restricted interests domain score, *ADI-D* ADI-before 36 months symptoms score, *ADOS-CSS* ADOS-Calibrated severity score

*Chi-squared value

Concerning first-degree relatives and TD, the presence of autistic symptoms was assessed by using the Social Responsiveness Scale-II (SRS-II) [14]. We also explored their Axis I psychiatric conditions (in accordance with DSM-IV-TR/5 criteria) using semi-standardized direct interviews, the Schedule for Affective Disorders and Schizophrenia for School-Age Children, Present and Lifetime version (K-SADS-PL) [33] for subjects below 18-year-old or the Diagnostic Interview for Genetic Studies (DIGS) [50] for adults. The non-verbal cognitive abilities of first-degree relatives and TD were estimated with Raven’s Progressive Matrices (RPM) [52]. Those with low, mild or below intellectual disabilities were excluded (IQ < 70).

### Exploration of RRBI

To explore the diversity of RRBI in all subjects in the study, we used the Repetitive Behavior Scale-Revised (RBS-R) [35] and the Yale-Brown Obsessive Compulsive Scale (Y-BOCS) [23], both considered as gold standard questionnaires to explore repetitive/stereotyped behaviors and obsessive–compulsive symptoms, respectively. The use of these 2 scales simultaneously and whatever the clinical profile of individuals we investigated allowed the exploration of the wide span of RRBI. For the purpose of the study, we used a self-report version of the Y-BOCS designed by the Tourette Syndrome Association Genetic Consortium (January 1995) [23]. This instrument, based on the symptom checklist and ordinal scales of the Y-BOCS, was used as parental hetero-questionnaires for children and as self-questionnaires for adults. The concordance for expert clinicians' assessment of obsessive–compulsive symptom severity was excellent [61].

### Brain volume estimations based on magnetic resonance imaging

MRI data were collected for a subsample of the subjects included in the study (*n *= 152), gathering 42 individuals with ASD, 89 first-degree relatives and 13 subjects from the general population (Additional file 1: Table S1). Acquisitions were performed using the following parameters: spoiled gradient recalled echo (SPGR), 1 mm isotropic, repetition time (TR) = 25 ms, echo time (TE) = 6 ms, flip angle = 30º. For all participants, MRI data were collected using previously described parameters, with a 1.5 Tesla scanner using a T1-Weighted acquisition [44]. Raw DICOM images were converted to NIFTI format with dcm2niix (https://github.com/rordenlab/dcm2niix) and defaced with MRIdeface [5]. Cortical reconstruction and volumetric segmentation were performed with FreeSurfer software version 6.0.0 (http://surfer.nmr.mgh.harvard.edu/), and visual control of the segmentation quality was done using the QCAPP (https://github.com/neuroanatomy/QCApp).

### Statistical Analysis

The demographic and clinical characteristics of the three groups of participants (autistic individuals, their first-degree relatives and TD) were compared using the Student’s *t* test and the *X*^2^ test for continuous and discrete variables, respectively. To investigate the dimensions underlying RRBI variability, we performed a principal component analysis (PCA). We first standardized item-related scores from the RBS-R and the Y-BOCS. We then ran general linear models adjusted for age, sex, and age*sex interactions to generate residuals (Additional file 1: Tables S2 and S3). PCA with varimax rotation was then performed on the residual standardized RRBI values, including all the participants enrolled in the study regardless of their status. The number of factors was defined after visual exploration of the screen plot. To identify the items belonging to a specific factor, we observed which items had high loadings (> |0.20|) for a specific factor but low loadings (< |0.10|) for the others. PCA was performed using JMP Pro 16.0 (SAS Inc., Cary, NC).

To explore the interactions between RRBI-derived factors and brain volumes, we ran a similar analysis on brain volumes extracted from automatic segmentation of the gray and white matter structures. Each brain volume was standardized. We also used general linear models adjusted for age, sex, and age*sex interactions to generate structural brain volume residuals. The relationship between the RRBI-derived factors and standardized structural brain volume residuals was explored using linear regression methods. All tests were two-tailed. Type I error rate was controlled using the false discovery rate (FDR) method. Statistical analysis was performed using Python packages (Python Software Foundation. Python Language Reference, version 2.7). Brain map figures were generated using the ENIGMA toolbox [38].

## Results

### Dimensionality of the RRBI symptoms

To explore the main dimensions underlying the diversity of RRBI reported in subjects enrolled in the study, we ran a principal component analysis. After visual inspection, we identified 3 main factors which accounted for 30.3% of the cumulative variance (Additional file 1: Table S4). Factor 1 (FA1, 12.7% of the variance) reflecting the ‘motor-driven’ RRBI or ‘sensory motor behaviors / compulsions’, with 43 items belonging to the RBS-R scale except one from the Y-BOCS. This item reflected the need to repeat routine activities—a frequent symptom reported in autistic patients, often associated with emotional dysregulation. In contrast, Factors 2 and 3 gathered mainly Y-BOCS-related items, reflecting the ‘cognitively driven’ RRBI. Factor 2 encompassed 22 items, all from the Y-BOCS (FA2, 8.8% of the variance). FA2 gathered symmetry and ordering symptoms that may represent a ‘rigidity/insistence on sameness’ dimension. Factor 3 aggregated items associated with washing, checking, contamination and aggressive obsessive–compulsive symptoms (FA3, 7.9% of the variance). The subjects’ scores showed a similar repartition depending on their conditions (autistic, related or TD) among the 3 factors (Additional file 3: Fig. S2).

#### Correlates between structural brain volumes and RRBI-related dimensions

**Fig. 1:**
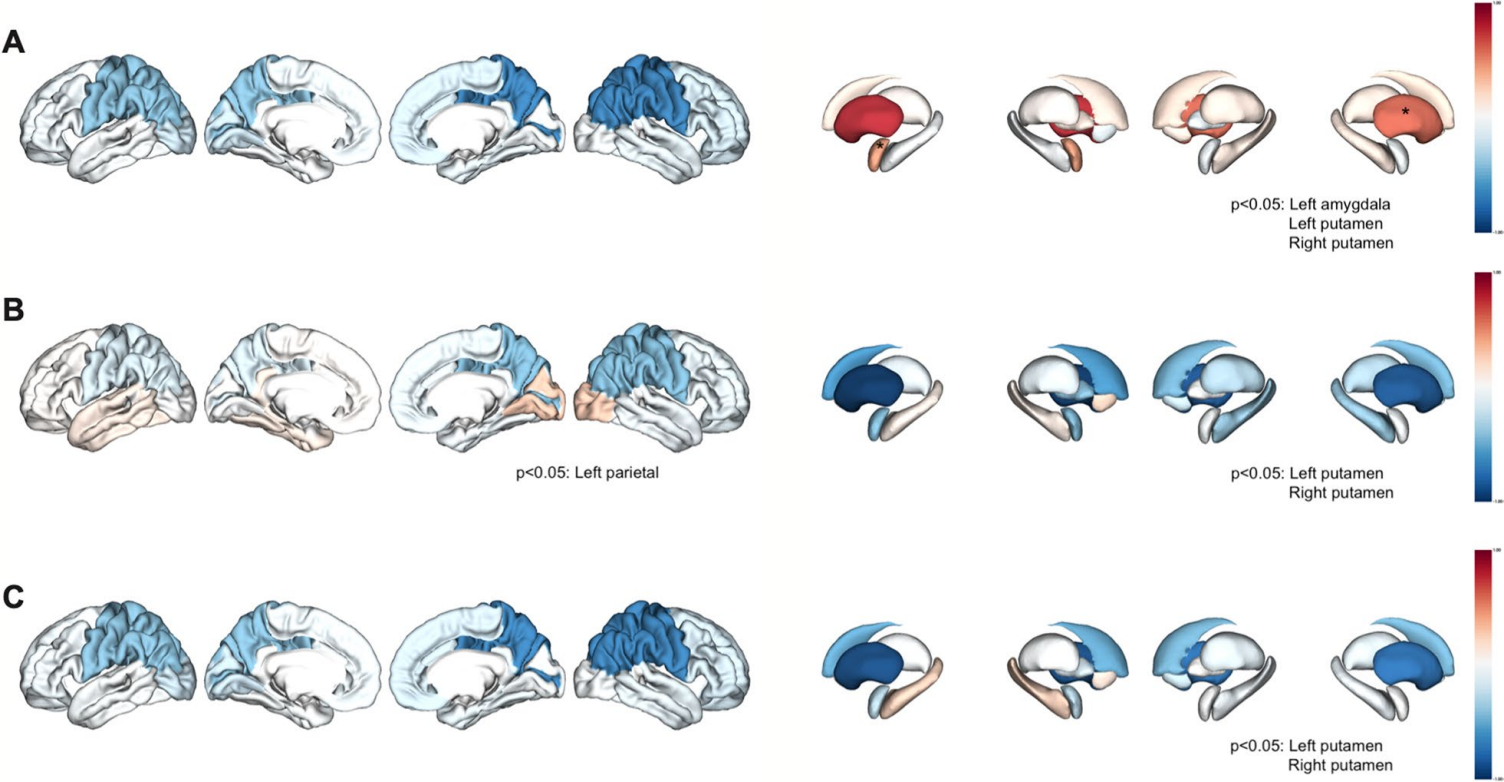
Brain map of linear regression-derived coefficients regarding the relationship between repetitive and restricted behaviors and interests-related factors and neuroanatomic structures. **A** Representation of the linear regression-derived coefficient obtained for each cortical volume (at left) and subcortical volume (at right) considering Factor 1. Extreme values of the linear regression-derived coefficients are represented in blue (<  − 1) and in red (> + 1). *P* values < 0.05 before FDR correction were obtained for the left amygdala, left putamen and right putamen volumes; **B** Representation of the linear regression-derived coefficient obtained for each cortical volume (at left) and subcortical volume (at right) considering Factor 2. Extreme values of the linear regression-derived coefficients are represented in blue (<  − 1) and in red (> + 1). *P* values < 0.05 before FDR correction were obtained for the left parietal, left putamen and right putamen volumes; **C** Representation of the linear regression-derived coefficient obtained for each cortical volume (at left) and each subcortical volume (at right) considering Factor 3. Extreme values of the linear regression-derived coefficients are represented in blue (<  − 1) and in red (> + 1). *P* values < 0.05 before FDR correction were obtained for the left amygdala, left putamen and right putamen volumes

We then explored the relationships between the RRBI-related dimensions and structural brain volume residuals using simple linear regressions (Fig. 1, Table 2, Additional file 2: Fig. S1). FA1 was associated with increased left and right putamen volumes (*R*^2^ = 0.06, *F *= 8.84, regression coefficient = 0.75, uncorrected *p *< 10^–3^, *r *= 0.24; *R*^2^ = 0.04, *F *= 5.48, regression coefficient = 0.58, uncorrected *p *< 10^–3^, *r *= 0.20). An interaction between FA1 and the left amygdala volume was also observed (*R*^2^ = 0.03, *F *= 4.18, regression coefficient = 0.03, uncorrected *p *= 0.04, *r *= 0.17). Our results were coherent with a recurrently reported brain volume increase in the amygdala in individuals with autism or anxiety disorders. However, the associations we reported did not survive adjustment for multiple comparisons (FA1 and left amygdala volume: corrected *p *= 0.41; FA1 and left putamen volume: corrected *p *= 0.10, FA1 and right putamen volume: corrected *p *= 0.30) (Fig. 1A, Table 2).

**Table 2:** Linear regressions between the factors and each cortical, subcortical, cerebellar and callous structures

	Factor 1					Factor 2					Factor 3				
	*F*	*R*^2^	Coef	*p*	FDR	*F*	*R*^2^	Coef	*p*	FDR	*F*	*R*^2^	Coef	*p*	FDR
*Cortical structures*															
Left frontal	0.09	0.001	0.12	0.75	0.91	0.003	0.0001	0.03	0.99	0.99	0.04	0.0001	− 0.10	0.85	0.97
Right frontal	0.23	0.002	0.17	0.63	0.90	0.05	0.0001	− 0.12	0.82	0.99	0.17	0.001	− 0.21	0.68	0.97
Left occipital	0.52	0.004	0.16	0.47	0.90	0.0003	0.0001	− 0.01	0.99	0.99	0.49	0.003	− 0.23	0.49	0.97
Right occipital	0.94	0.007	0.22	0.33	0.90	0.51	0.004	0.26	0.48	0.87	0.03	0.0001	− 0.05	0.87	0.97
Left parietal	0.29	0.002	0.16	0.59	0.90	0.16	0.001	− 0.18	**0.001**	0.10	0.95	0.007	− 0.43	0.33	0.97
Right parietal	1.37	0.01	0.34	0.24	0.90	0.98	0.007	− 0.45	0.32	0.86	2.66	0.02	− 0.69	0.11	0.44
Left temporal	1.02	0.007	0.28	0.31	0.90	0.08	0.001	0.12	0.78	0.99	0.01	0.0001	− 0.04	0.93	0.97
Right tempora	0.64	0.005	0.21	0.42	0.90	0.06	0.0001	− 0.09	0.83	0.99	0.09	0.001	− 0.12	0.76	0.97
*Subcortical structures*															
Left accumbens	0.25	0.002	− 0.09	0.62	0.90	0.54	0.004	0.21	0.46	0.87	0.23	0.002	0.13	0.64	0.97
Right accumbens	0.32	0.002	0.11	0.57	0.90	0.58	0.004	− 0.22	0.45	0.87	0.63	0.005	− 0.22	0.43	0.97
Left amygdala	**4.18**	0.030	0.41	**0.04**	0.41	1.89	0.01	− 0.44	0.17	0.75	0.80	0.006	− 0.27	0.37	0.97
Right amygdala	0.10	0.001	− 0.06	0.76	0.91	0.02	0.0001	− 0.04	0.89	0.75	0.002	0.0001	− 0.01	0.97	0.97
Left caudate	0.38	0.003	0.13	0.54	0.90	3.39	0.02	− 0.61	0.07	0.66	2.94	0.02	− 0.53	0.89	0.97
Right caudate	0.64	0.43	0.16	0.43	0.90	2.25	0.02	− 0.48	0.14	0.75	2.65	0.02	− 0.48	0.11	0.44
Left hippocampus	0.02	0.0001	− 0.03	0.89	0.92	0.08	0.001	0.09	0.78	0.98	0.51	0.004	0.23	0.47	0.97
Right hippocampus	0.35	0.002	0.12	0.55	0.90	1.48	0.01	− 0.39	0.23	0.78	0.07	0.001	− 0.08	0.79	0.97
Left pallidum	0.21	0.002	0.13	0.64	0.90	1.04	0.008	− 0.43	0.31	0.86	0.25	0.002	− 0.19	0.62	0.97
Right pallidum	0.17	0.001	− 0.10	0.68	0.90	0.003	0.0001	− 0.02	0.96	0.99	0.004	0.0001	0.02	0.95	0.97
Left putamen	**8.84**	0.06	**0.75**	**0.003**	0.10	**8.06**	0.06	** − 1.12**	**0.005**	0.15	**6.20**	0.04	** − 0.92**	**0.01**	0.41
Right putamen	**5.48**	0.04	**0.58**	**0.02**	0.30	**5.31**	0.04	** − 0.89**	**0.02**	0.33	**4.60**	0.32	** − 0.78**	**0.03**	0.44
Left thalamus	0.04	0.0001	0.05	0.84	0.92	0.08	0.001	− 0.11	0.78	0.99	0.005	0.0001	− 0.03	0.94	0.97
Right thalamus	0.20	0.001	0.11	0.65	0.90	0.58	0.004	− 0.29	0.45	0.86	0.12	0.001	− 0.12	0.73	0.97
*Cerebellum*															
Left Cerebellum	0.01	0.01	0.27	0.19	0.90	1.38	0.01	− 0.37	0.24	0.78	1.03	0.007	− 0.29	0.31	0.97
Right Cerebellum	1.33	0.01	0.24	0.25	0.90	0.83	0.006	− 0.30	0.36	0.87	0.66	0.005	− 0.24	0.42	0.97
*Corpus Callosum*															
Anterior corpus callosum	1.57	0.001	− 0.004	0.19	0.98	0.01	0.0001	0.02	0.93	0.99	0.13	0.001	− 0.09	0.72	0.97
Mid anterior corpus callosum	0.02	0.001	− 0.02	0.89	0.92	0.04	0.0001	0.05	0.84	0.99	0.09	0.001	− 0.07	0.77	0.97
Central corpus callosum	0.33	0.001	0.56	0.56	0.90	0.30	0.002	0.14	0.59	0.99	0.12	0.001	0.08	0.73	0.97
Midposterior corpus callosum	0.57	0.004	0.14	0.45	0.90	2.39	0.02	− 0.43	0.13	0.75	2.98	0.02	− 0.44	0.08	0.44
Posterior corpus callosum	0.02	0.001	0.03	0.89	0.92	1.81	0.01	− 0.46	0.18	0.75	2.89	0.02	− 0.54	0.09	0.44

*F* value, *R*-squared, linear regression-derived coefficients (Coef), standard errors (SE) and *p* value from the linear regressions were reported; FDR: False Discovery Rate was calculated to correct for multiple testing. In bold, non-corrected *p* value < 0.05

FA2 was also associated with decreased left and right putamen volumes (respectively, *R*^2^ = 0.06, *F *= 8.06, regression coefficient =  − 1.12, uncorrected *p *< 10^–3^, *r *=  − 0.24; *R*^2^ = 0.04, *F *= 5.31, regression coefficient =  − 0.89, uncorrected *p *= 0.02, *r *=  − 0.19) and decreased left parietal volume (*R*^2^ = 0.001, *F *= 0.16, regression coefficient =  − 0.18, uncorrected *p *< 10^–3^, *r *=  − 0.05). Interestingly, decreased volume in the parietal lobe was associated in the literature with impairment in high-order cognitive processes, mainly executive dysfunctions. Despite the relevance of our findings, their significance did not survive after multiple comparisons correction (FA2 and left putamen volume: corrected *p *= 0.15; FA2 and right putamen volume: corrected *p *= 0.33; FA2 and left parietal volume: corrected *p *= 0.10) (Fig. 1B and Table 2). Similarly, FA3 was linked to decreased left and right putamen volumes (*R*^2^ = 0.04, *F *= 6.20, coefficient =  − 0.92, uncorrected *p *= 0.01,  − 0.21; *R*^2^ = 0.32, *F *= 4.60, coefficient =  − 0.78, uncorrected *p *= 0.03, *r *=  − 0.18). This association did not persist after correction for multiple comparisons (FA3 and left putamen volume: corrected *p *= 0.41; FA3 and right putamen volume: corrected *p *= 0.44) (Fig. 1C and Table 2).

## Discussion

The challenges in identifying biomarkers in neurodevelopmental disorders, particularly in autism, stem from the difficulties researchers have in considering the inter-individual phenotypic variability. It ranges from those with a developmental trajectory in the normal range (although carrying environmental and/or genetic vulnerability risk factors) to individuals with mild to severe impairment. The purpose of our study was, therefore, to account for this inter-individual variability by exploring the spectrum of RRBI assessed in a large population of individuals with autism, their unaffected first-degree relatives, and controls from the general population. Interestingly, our results underlined the potential implication of the putamen in the RRBI: an increase in the volume of the putamen was associated with 'low-order' symptoms (mainly stereotyped behaviors) and on the opposite, a decrease in the putamen volume with cognitively driven symptoms (mainly obsessive–compulsive symptoms).

### Dimensionality of the RRBI symptoms

The factor analysis revealed the multidimensionality of RRBI by identifying three dimensions: FA1 gathered the RBS-R-related items typically observed in autism [13, 32]; FA2 put together the items related to rigidity/insistence on sameness from the Y-BOCS, symptoms which were trait features of the individuals with autism but also those with obsessive–compulsive symptoms [65, 68]; and FA3 that included only Y-BOCS related items usually displayed by individuals with OCD [17]. Interestingly, FA2 put together the symmetry and ordering of obsessions/compulsions and the need to repeat things. These symptoms were described in the literature as settled clinical characteristics of autistic children with comorbid OCD [55]. Symmetry and ordering symptoms are frequently reported in the developmental subtype of OCD, which—beyond its juvenile onset—is characterized by an increased proportion of neurodevelopmental comorbidities, including Tourette's syndrome, ADHD, and autism, but also by significant impairment in executive functions [11, 16, 24]. A few studies have highlighted the link between the intensity of symmetry/ordering symptoms and poor verbal working memory, visuospatial planning, inhibitory control, and cognitive flexibility abilities [11]. Thus, among the characteristics shared by children with autism and OCD, executive dysfunction may play a critical role in these disorders [10, 30]. Interestingly, unaffected first-degree parents of children with OCD or autism also showed executive impairments [17, 47, 59]. Overall, FA2 dimension we identified may be a testimony of the cumulative impact of executive impairment and the presence of RRBI. Finally, the FA3 dimension was driven by OCD-related symptoms since gathering the washing, checking, contamination, and aggressive symptoms [36, 55]. This dimension was probably less related to autism but more a proxy of obsessive–compulsive symptoms [40, 45].

#### Correlation between structural brain volumes and RRBI-related dimensions

Our results were in line with publications stressing the critical role of the cortico-thalamic-striatal-cortical loop in RRBI. Our study revealed a central implication of the putamen across the 3 distinct dimensions we reported, as well as specific roles of subcortical and cortical structures per FA, which may shape the diversity of symptoms, agglomerated on the dimensions. The positive association of the putamen and the left amygdala with FA1 was consistent with the role of the putamen in autonomic movements, described in complex motor stereotypies [43] and in autism [19, 37, 46, 53]. Including the putamen, the basal ganglia play important roles in regulating repetitive behaviors (notably in autism) in association with the hippocampus, the hypothalamus, but also other neuroanatomical structures of the limbic system (including the amygdala) [22]. Obviously, amygdala volume abnormalities were more usually related to anxiety, and more specifically to social anxiety in autism [3, 22, 31].

In contrast to the positive association of the putamen volume with FA1, we observed a negative relationship with FA2 and FA3. Interestingly, our results replicated those of the ENIGMA-OCD consortium, which also reported a reduced putamen volume in OCD [62, 63]. This reduced putamen volume may relate more to the compulsive component than the obsessive one in OCD [9]. FA2 was associated in addition to the decreased volume of the parietal cortex. Similar parietal abnormalities in cortical thickness, volume or surface area, may reflect the cortical dysmaturation in this area, frequently reported in children with OCD and autism [6, 53, 54]. Interestingly, the parietal cortex is involved in social interactions, motor learning and repetitive behaviors in autism [64] but also participates in cognitive in/flexibility, which is coherent with the sameness nature of RRBI-related symptoms encompassed by FA2 [26]. Bidirectional associations between RRBI and putamen volume highlighted its pivotal role, implying the presence of modulators beyond the putamen. Although FA1, FA2 and FA3 were distinct factors, shared neural underpinnings might contribute to their clinical similarity.

### Limitations

Our study was conducted retrospectively in relation to the initial research project using a 1.5 T MRI and FreeSurfer, which reduced the data acquisition and segmentation precision of the brain structure, especially the subcortical ones such as the putamen. Nevertheless, we maintained the quality of FreeSurfer segmentations through a visual quality check in our study. Furthermore, the use of FreeSurfer segmentation facilitated comparisons with large cohorts such as ENIGMA, enhancing the relevance of our results. The lack of power of the brain imaging part of our study did not allow us to explore the brain asymmetry structures associated with the dimensions we reported. We, however, observed a trend for a leftward asymmetry (based on *p* value and coefficients *L* > *R*). A similar leftward brain asymmetry involving the putamen was previously reported in autism and OCD [7, 28, 34, 51, 54]. The role and impact of this brain asymmetry on the symptomatology of ASD and OCD remained unclear but may result from aberrant brain development trajectories.

When exploring the dimensions gathering the RRBI phenotypic variability, we did not investigate the gender’s impact on RRBI due to the limited sample size of females, which was a significant limitation [1, 2, 21, 60]. Nevertheless, we conducted sub-analyses exclusively on male subjects, maintaining the consistent item composition of the three FAs. (Additional file 1: Table S5). We also did not include the effect of potential covariates, such as the whole pattern of comorbidities or the effect of executive dysfunctions, as mentioned above. For example, planning strategy impairments consistently reported in probands, and their first-degree relatives may specifically participate in FA2 [8]. Based on scales (RBS-R and Y-BOCS), respectively, dedicated to repetitive behaviors in autism and obsessive–compulsive symptoms, our study was limited by their own constructions, which impacted on the dimensional approach. Future research would explore whether similar dimensions emerge when using a single integrated scale [48].

Finally, one additional limitation may result from the wide range of ages of individuals enrolled in our study. This might have biased the volume estimates of the small brain structures, specifically subcortical structures such as the putamen or the amygdala, since it relied on a limited number of voxels [27, 42, 57]. This effect may result from the opposite coefficient direction of the regression analysis, we reported between FA1 and the neuroanatomical structures (positive regression) and between FA2 or FA3 (negative regression) and these. FA1 more reflected the autistic-related symptoms and thus was mainly based on symptoms displayed by probands with autism, younger than relatives and controls enrolled in our study. Moreover, our study was based on cross-sectional, not longitudinal, which limited the exploration of changes in trajectories reported in autism and OCD [25, 41, 49].

## Conclusion

Our results stressed the pivotal role of the putamen in the determinism of RRBI. Variations in the putamen volume, in association with variations in cortical structures, influence the phenomenology of repetitive behaviors in individuals with autism, their relatives, and individuals from the control population. Exploration of the integrative role of the putamen needs to be strengthened in this specific context.

### Supplementary Information

**Additional file1: Table S1.** Clinical and demographic characteristics of the individuals enrolled in the study with MRI data. **Table S2.** Yale-Brown Obsessive Compulsive Scale Symptom Checklist Category Residuals Normalized Scores (Data are mean ± SD). **Table S3.** Repetitive Behaviors Scale - Revised Residuals Normalized Scores (Data are mean ± SD). **Table S4.** Item loading for the three factors yielded an analysis factor for the items of the Y-BOCS and the RBS-R, and domains related to each item among all participants. **Table S5. **Item loading for the three factors yielded an analysis factor for the items of the Y-BOCS and the RBS-R, and domains related to each item among male participants.

**Additional file 2: Fig. S1.** Linear regressions between repetitive and restricted behaviors and interests-related factors and the neuroanatomic volumes with a high absolute coefficient and a non-corrected *p* value<0.05. At the top, the figures represent the regressions between Factor 1 and the left putamen volume (at the left), the right putamen volume (in the middle) and the left amygdala volume (at the right). At the middle, the figures represent the regressions between Factor 2 and the left putamen volume (at the left), the right putamen volume (in the middle) and the left parietal volume (at the right). At the bottom, the figures represent the regressions between Factor 3 and the left putamen volume and the right putamen volume. The figures show an outsider to FA values. Excluding this outsider, the linear regressions between the three FA and the left and right putamen structures maintain a non-corrected *p* value<0.05, not between Factor 1 and the left amygdala volume, nor between Factor 2 and the left parietal volume.

**Additional file 3: Fig. S2.** Boxplots representing the distribution of individual scores by group [autistic (in blue), relatives (in orange) and controls (in red) groups] for FA1 (at the left), FA2 (in the middle), FA3 (at the right).

## Data Availability

The datasets generated and/or analyzed during the current study are not publicly available due to an embargo period but are available from the corresponding author on reasonable request.

## Abbreviations

ADI-R: Autism diagnosis interview-revised
ADHD: Attention deficit hyperactivity disorder
ADOS-2: Autism diagnostic observation scale second edition
ADOS-2 CSS: Autism diagnostic observation schedule -second edition calibrated severity score
ASD: Autism spectrum disorder
DSM: Diagnostic and statistical manual of mental disorders
K-SADS-PL: Kiddie-schedule for affective disorders and schizophrenia for school age children, present and lifetime version
FA: Factor analysis
IQ: Intellectual quotient
OCD: Obsessive–compulsive disorder
MRI: Magnetic resonance imaging
PARIS: Paris autism research international sib-pair
RRBI: Repetitive and restricted behaviors and interests
RPM: Raven’s progressive matrices
TD: Typically developing
