# Supplementary material for: Exploring the multidimensional nature of repetitive and restricted behaviors and interests (RRBI) in autism: neuroanatomical correlates and clinical implications
Source: Mol Autism. 2023 Nov 27;14:45. doi: 10.1186/s13229-023-00576-z (PMC10680239; doi:10.1186/s13229-023-00576-z)
Supplement: Supplementary file 1 — Additional file1: Table S1. Clinical and demographic characteristics of the individuals enrolled in the study with MRI data. Table S2. Yale-Brown Obsessive Compulsive Scale Symptom Checklist Category Residuals Normalized Scores (Data are mean ± SD). Table S3. Repetitive Behaviors Scale - Revised Residuals Normalized Scores (Data are mean ± SD). Table S4. Item loading for the three factors yielded an analysis factor for the items of the Y-BOCS and the RBS-R, and domains related to each item among all participants. Table S5. Item loading for the three factors yielded an analysis factor for the items of the Y-BOCS and the RBS-R, and domains related to each item among male participants. [file 13229_2023_576_MOESM1_ESM.docx]

**SUPPLEMENTARY MATERIAL**

**Exploring the Multidimensional Nature of Repetitive and Restricted Behaviors and Interests (RRBI) in Autism: Neuroanatomical Correlates and Clinical Implications**

Aline Lefebvre^1,2,6*^, Nicolas Traut^3^, Amandine Pedoux^4^, Anna Maruani^2,4^, Anita Beggiato^2,4^, Monique Elmaleh^5^, David Germanaud^6,7,8^, Anouck Amestoy^9,10^, Myriam Ly-Le Moal^11^, Christopher Chatham^12^, Lorraine Murtagh^12^, Manuel Bouvard^9,10^, Marianne Alisson^5^, Marion Leboyer^13,14^, Thomas Bourgeron^2,16^, Roberto Toro^3^, Guillaume Dumas^17^, Clara Moreau^2,18^, Richard Delorme^1,2,10,16^

^1^ University Hospital of Child and Adolescent Psychiatry, Fondation Vallée, GHT Paris Sud, Gentilly, France

^2^ Human Genetics and Cognitive Functions, Institut Pasteur, UMR 3571 CNRS, University Paris Diderot, Paris, France

^3^ Institut Pasteur, Université Paris Cité, Unité de Neuroanatomie Appliquée et Théorique, Paris, France

^4^ Department of Child and Adolescent Psychiatry, Robert Debré Hospital, APHP, Paris, France

^5^ Department of Pediatric Radiology, APHP, Robert-Debré Hospital, Paris, France

^6^ UNIACT Neurospin CEA - INSERM UMR 1129 Université Paris Cité

^7^  Department of Clinical Genetics, Robert Debré Hospital, APHP, Paris, France

^8^ Center for Research and Interdisciplinarity (CRI), Université Paris Cité, Paris, France

^9^ Autism Expert Centre, Charles Perrens Hospital, Bordeaux, France

^10^ Fondation FondaMental, French National Science Foundation, Créteil, France

^11^ Institut Roche, Boulogne-Billancourt, France

^12^Roche Pharma Research and Early Development, Neuroscience and Rare Diseases, Roche Innovation Center Basel, F. Hoffmann–La Roche Ltd., Basel, Switzerland

^13^ Fondation FondaMental, French National Science Foundation, Créteil, France

^14^ Institut National de la Santé et de la Recherche Médicale (INSERM), U955, Institut Mondor de Recherche Biomédicale, Psychiatrie Translationnelle, Créteil, France

^15^ Department of Adult Psychiatry, Henri Mondor and Albert Chenevier Hospital, Créteil, France

^16^ Université Paris Cité

^17^ Department of Psychiatry, Université de Montreal, CHU Ste Justine Hospital, Montreal, QC, Canada

^18^ Imaging Genetics Center, Stevens Neuroimaging and Informatics Institute, Keck School of Medicine of USC, California, USA

***Corresponding author:** Aline Lefebvre, M.D., Human Genetics and Cognitive Functions, Institut Pasteur, 25 rue du Docteur Roux, 75015 Paris, France. Tel: + 33140613311. Fax: +33140613953. E-mail: [aline.lefebvre@ch-fondationvallee.fr](mailto:aline.lefebvre@ch-fondationvallee.fr)

**SUPPLEMENTARY TABLES**

**Supplementary Table 1.** Clinical and demographic characteristics of the individuals enrolled in the study with MRI data

|  | **ASD**  **(N=42)** | **Relatives (N=89)** | **Controls (N=13)** |  |  |  |  |
| --- | --- | --- | --- | --- | --- | --- | --- |
| **Age** | 12.9 (10.3) | 36.1 (13.3) | 19.1 (14.7) |  | 0.42 | 51.7 | <0.0001 |
| **Sex - Male ratio (%, n)** | 86% (36) | 41% (37) | 46% (6) |  | Chi2= 23.9 | - | <0.0001 |
| **NVIQ** | 100.2 (23.7) | 114.5 (15.4) | 111.6 (17.1) |  | 0.12 | 5.3 | 0.007 |
| **SRS T-score** | 76.7 (13.5) | 46.1 (8.8) | 46.3 (7.9) |  | 0.65 | 125.1 | <0.0001 |
| **ADI - A** | 18.0 (8.4) | - | - |  | - | - | - |
| **ADI - B** | 14.0 (7.1) | - | - |  | - | - | - |
| **ADI - C** | 5.8 (3.8) | - | - |  | - | - | - |
| **ADI - D** | 2.7 (1.6) | - | - |  | - | - | - |
| **ADOS-CSS** | 4.77 (1.84) | - | - |  | - | - | - |
| *ASD: autistic individuals; NVIQ: Non-verbal IQ; ADI-A: ADI-Social interaction domain score; ADI-B: ADI-Communication domain score; ADI-C: Stereotypes & restricted interests domain score; ADI-D: ADI-before 36 months symptoms score; ADOS-CSS: ADOS-Calibrated severity score; *Chi-squared value.* | | | | | | | |

**Supplementary Table 2.** Yale-Brown Obsessive-Compulsive Scale Symptom Checklist Category Residuals Normalized Scores (Data are mean ± SD)

|  | |  | | ASD individuals | Relatives | TD individuals | F (p-value) |
| --- | --- | --- | --- | --- | --- | --- | --- |
| **Obsessive symptoms** | | | | |  |  |  |
|  |  | | Aggressive | 0.18 (1.16) | -0.08 (0.86) | -0.37 (0.65) | 16.82 (<0.0001) |
|  |  | | Contamination | 0.13 (1.10) | -0.10 (0.86) | -0.31 (0.65) | 12.04 (<0.0001) |
|  |  | | Sexual | 0.06 (1.08) | -0.09 (0.86) | -0.19 (0.62) | 4.31 (0.01) |
|  |  | | Hoarding | 0.11 (1.08) | -0.06 (0.94) | -0.26 (0.71) | 7.60 (0.0005) |
|  |  | | Religious | 0.07 (0.98) | -0.05 (0.93) | -0.4 (0.44) | 15.18 (<0.0001) |
|  |  | | Symmetry | 0.26 (1.12) | -0.18 (0.84) | -0.4 (0.49) | 32.01 (<0.0001) |
|  |  | | Somatic | 0.09 (1.06) | -0.04 (0.95) | -0.34 (0.69) | 10.46 (<0.0001) |
| **Compulsive symptoms** | | | | |  |  |  |
|  |  | | Cleaning | 0.12 (1.01) | -0.24 (0.62) | -0.35 (0.46) | 25.21 (<0.0001) |
|  |  | | Checking | 0.11 (1.18) | -0.03 (0.91) | -0.45 (0.58) | 17.28 (<0.0001) |
|  |  | | Repeating | 0.18 (1.05) | -0.25 (0.68) | -0.45 (0.43) | 37.35 (<0.0001) |
|  |  | | Counting | 0.21 (1.17) | -0.20 (0.75) | -0.35 (0.44) | 25.09 (<0.0001) |
|  |  | | Ordering | 0.31 (1.12) | -0.19 (0.83) | -0.41 (0.61) | 36.71 (<0.0001) |
|  |  | | Hoarding | 0.26 (1.20) | -0.19 (0.76) | -0.27 (0.62) | 24.27 (<0.0001) |

*ASD: Autism Spectrum Disorder; TD: Typically developing; Data are means (+/- standard deviation).*

**Supplementary Table 3.** Repetitive Behaviors Scale - Revised Residuals Normalized Scores (Data are mean ± SD)

|  | ASD individuals | Relatives | TD individuals | F (p-value) |
| --- | --- | --- | --- | --- |
| Stereotypy Subscale | 0.76 (1.29) | -0.38 (0.50) | -0.41 (0.33) | 150.9 (<0.0001) |
| Self-injurious Subscale | 0.46 (1.46) | -0.21 (0.55) | -0.26 (0.46) | 42.22 (<0.0001) |
| Compulsive Subscale | 0.60 (1.31) | -0.27 (0.66) | -0.37 (0.41) | 79.64 (<0.0001) |
| Ritualistic Subscale | 0.76 (1.29) | -0.37 (0.46) | -0.43 (0.39) | 151.14 (<0.0001) |
| Sameness Subscale | 0.78 (1.27) | -0.36 (0.50) | -0.48 (0.33) | 160.49 (<0.0001) |
| Restricted Subscale | 0.87 (1.23) | -0.47 (0.28) | -0.40 (0.53) | 225.43 (<0.0001) |

*ASD: Autism Spectrum Disorder; TD: Typically developing; Data are means (+/- standard deviation).*

**Supplementary Table 4.** Item loading for the three factors yielded an analysis factor for the items of the Y-BOCS and the RBS-R, and domains related to each item among all participants

| **Scale** | **Domain** | **Item** | **Factor loadings**  **(% of variance)** | | |
| --- | --- | --- | --- | --- | --- |
|  |  |  | **Factor 1 (12.7%)** | **Factor 2 (8.8%)** | **Factor 3 (7.9.%)** |
| RBS-R | Compulsive scale | 21. Repeating | **0.71** | -0.05 | -0.01 |
| RBS-R | Compulsive scale | 22. Needs to touch | **0.66** | 0.03 | -0.06 |
| RBS-R | Ritualistic Subscale | 25. Self care routine | **0.65** | 0.05 | 0.23 |
| RBS-R | Stereotypy subscale | 6. Sensory | **0.65** | 0.16 | -0.08 |
| RBS-R | Stereotypy subscale | 3. Finger movements | **0.64** | 0.32 | -0.14 |
| RBS-R | Compulsive scale | 18. Checking | **0.62** | 0.14 | -0.09 |
| RBS-R | Sameness Subscale | 38. Insists on routine | **0.61** | 0.02 | 0.25 |
| RBS-R | Compulsive scale | 16. Completeness | **0.61** | 0.10 | -0.11 |
| RBS-R | Stereotypy subscale | 2. Head movements | **0.60** | 0.08 | -0.02 |
| RBS-R | Compulsive scale | 19. Counting | **0.59** | 0.09 | 0.04 |
| RBS-R | Ritualistic Subscale | 24. Sleeping/bedtime | **0.59** | 0.01 | 0.09 |
| RBS-R | Sameness Subscale | 30. No new places | **0.58** | 0.04 | 0.18 |
| RBS-R | Sameness Subscale | 33. Sits certain place | **0.58** | 0.00 | 0.25 |
| RBS-R | Ritualistic Subscale | 23. Eating/mealtime | **0.57** | 0.05 | 0.00 |
| RBS-R | Stereotypy subscale | 5. Object usage | **0.56** | 0.22 | -0.15 |
| RBS-R | Restricted Subscale | 40. Preoccupation with subject | **0.56** | 0.16 | 0.05 |
| RBS-R | Restricted Subscale | 43. Preoccupation with movement | **0.55** | 0.07 | -0.17 |
| RBS-R | Ritualistic Subscale | 26. Transportation routine | **0.54** | 0.13 | 0.05 |
| RBS-R | Sameness Subscale | 35. Uses certain door | **0.54** | -0.05 | 0.20 |
| RBS-R | Compulsive scale | 17. Washing | **0.54** | 0.17 | 0.01 |
| RBS-R | Sameness Subscale | 39. Insists on time | **0.54** | -0.01 | 0.21 |
| RBS-R | Sameness Subscale | 32. Walks certain way | **0.53** | 0.02 | -0.01 |
| RBS-R | Sameness Subscale | 31. No interruption | **0.53** | 0.19 | 0.08 |
| RBS-R | Ritualistic Subscale | 28. Communication | **0.52** | 0.03 | 0.14 |
| RBS-R | Sameness Subscale | 37. Difficult transitions | **0.51** | 0.28 | 0.20 |
| RBS-R | Self-injurious subscale | 13. Inserts finger/object | **0.49** | 0.00 | 0.00 |
| RBS-R | Self-injurious subscale | 14. Picks skin | **0.48** | 0.07 | 0.04 |
| RBS-R | Self-injurious subscale | 11. Pulls hair/skin | **0.47** | -0.05 | 0.03 |
| RBS-R | Stereotypy subscale | 1. Body movements | **0.46** | 0.08 | 0.08 |
| RBS-R | Restricted Subscale | 42. Preoccupied with part of object | **0.45** | 0.13 | 0.10 |
| RBS-R | Sameness Subscale | 36. Videotapes | **0.45** | 0.18 | 0.08 |
| RBS-R | Sameness Subscale | 29. Placements of objects | **0.45** | 0.27 | 0.16 |
| RBS-R | Compulsive scale | 20. Hoarding | **0.45** | 0.01 | 0.13 |
| RBS-R | Sameness Subscale | 34. Appearance/behavior of others | **0.44** | 0.20 | 0.22 |
| RBS-R | Ritualistic Subscale | 27. Play/leisure routine | **0.43** | 0.05 | 0.03 |
| RBS-R | Self-injurious subscale | 10. Bites self | **0.42** | 0.06 | 0.17 |
| Y-BOCS | Repeating Rituals | 13. Need to repeat routine activities | **0.41** | 0.23 | 0.03 |
| RBS-R | Restricted Subscale | 41. Attached to subject | **0.40** | 0.02 | 0.12 |
| RBS-R | Stereotypy subscale | 4. Locomotion | **0.39** | 0.21 | -0.08 |
| RBS-R | Self-injurious subscale | 9. Hits w/object | **0.38** | -0.07 | -0.02 |
| RBS-R | Self-injurious subscale | 12. Rubs/scratches | **0.38** | 0.02 | 0.07 |
| RBS-R | Self-injurious subscale | 8. Hits against surface | **0.31** | 0.06 | 0.29 |
| RBS-R | Self-injurious subscale | 7. Hits w/body | **0.31** | 0.12 | -0.09 |
| *RBS-R* | *Compulsive scale* | *15. Ordering* | *0.30* | *0.29* | *0.01* |
| *Y-BOCS* | *Miscellaneous Compulsions* | *21. Rituals involving blinking or staring* | *0.24* | *0.23* | *0.08* |
| Y-BOCS | Obsessions with need for symmetry or exactness | 67. Obsession with need to even things out | 0.03 | **0.71** | -0.19 |
| Y-BOCS | Obsessions with need for symmetry or exactness | 64. Obsessions with need for alignment | 0.03 | **0.71** | -0.19 |
| Y-BOCS | Ordering/Arranging Compulsions | 18. Need to make things even or balanced | 0.09 | **0.66** | -0.05 |
| Y-BOCS | Obsessions with need for symmetry or exactness | 61. Obsessions with need for exactness | 0.23 | **0.65** | 0.08 |
| Y-BOCS | Compulsions with need of symmetry/exactness | 17. Compulsions with need of symmetry/exactness | 0.14 | **0.59** | 0.05 |
| Y-BOCS | Ordering/Arranging Compulsions | 16. Ordering/arranging compulsions | 0.17 | **0.58** | 0.08 |
| Y-BOCS | Miscellaneous Compulsions | 20. Mental rituals | 0.00 | **0.58** | 0.23 |
| Y-BOCS | Obsessions with need for symmetry or exactness | 62. Obsessions with need for symmetry | 0.22 | **0.57** | 0.06 |
| Y-BOCS | Checking Compulsions | 11. Checking tied to somatic obsessions | 0.15 | **0.52** | 0.16 |
| Y-BOCS | Hoarding/Saving Obsessions | 58. Hoarding/saving obsessions | -0.01 | **0.50** | 0.37 |
| Y-BOCS | Miscellaneous Obsessions | 75. Bothered by certains sounds/noises | 0.27 | **0.47** | 0.28 |
| Y-BOCS | Checking Compulsions | 9. Checking that did not make mistake | 0.04 | **0.47** | 0.31 |
| Y-BOCS | Somatic Obsessions | 80. Excessive concern with body part or aspect of appearance | -0.03 | **0.46** | 0.26 |
| Y-BOCS | Repeating Rituals | 12. Re-reading or re-writing | 0.27 | **0.45** | 0.23 |
| Y-BOCS | Contamination Obsessions | 52. Concern with dirt or germs | 0.09 | **0.44** | 0.24 |
| Y-BOCS | Miscellaneous Obsessions | 74. Intrusive nonsense sounds words, or music | 0.08 | **0.44** | 0.33 |
| Y-BOCS | Miscellaneous Compulsions | 27. Ritualized eating behaviors | 0.15 | **0.41** | 0.07 |
| Y-BOCS | Counting Compulsions | 15. Counting compulsions | 0.23 | **0.40** | 0.08 |
| *Y-BOCS* | *Miscellaneous Obsessions* | *68. Need to know or remember* | *0.20* | *0.39* | *0.32* |
| Y-BOCS | Cleaning/Washing compulsions | 4. Cleaning of household items, inanimate objects or pets. | 0.03 | **0.39** | 0.07 |
| *Y-BOCS* | *Contamination Obsessions* | *56. Concerned will get ill because of contaminant* | *-0.01* | *0.37* | *0.34* |
| Y-BOCS | Miscellaneous Compulsions | 24. Need to touch, tap, or rub | 0.17 | **0.37** | 0.13 |
| *Y-BOCS* | *Miscellaneous Obsessions* | *73. Intrusive images* | *0.00* | *0.36* | *0.34* |
| Y-BOCS | Aggressive Obsessions | 37. Fear might harm self | -0.16 | **0.36** | 0.10 |
| Y-BOCS | Miscellaneous Obsessions | 72. Fear of losing things | 0.06 | **0.36** | 0.15 |
| Y-BOCS | Somatic Obsessions | 79. Concern with illness or disease | 0.08 | **0.35** | 0.21 |
| Y-BOCS | Checking Compulsions | 10. Others | 0.23 | **0.35** | 0.01 |
| *Y-BOCS* | *Hoarding / Collecting Compulsions* | *19. Hoarding/collecting compulsions* | *0.03* | *0.26* | *0.18* |
| Y-BOCS | Cleaning/Washing Compulsions | 1. Excessive or ritualized hygiene | 0.03 | **0.24** | 0.05 |
| *Y-BOCS* | *Miscellaneous Compulsions* | *36. Trichotillomania* | *0.01* | *0.13* | *0.08* |
| Y-BOCS | Checking Compulsions | 6. Checking that did not/will not harm others | 0.05 | 0.12 | **0.64** |
| Y-BOCS | Checking Compulsions | 7. Checking that did not/will not harm self | 0.23 | 0.13 | **0.62** |
| Y-BOCS | Aggressive Obsessions | 40. Fear of blurting out obscenities or insults | 0.17 | 0.10 | **0.62** |
| Y-BOCS | Aggressive Obsessions | 38. Fear might harm others | -0.02 | 0.16 | **0.61** |
| Y-BOCS | Contamination Obsessions | 53. Excessive concern with environmental contaminants | -0.05 | 0.20 | **0.57** |
| Y-BOCS | Miscellaneous Obsessions | 76. Lucky/unlucky numbers | 0.04 | 0.19 | **0.55** |
| Y-BOCS | Miscellaneous Compulsions | 28. Superstitious behaviors | 0.07 | -0.05 | **0.51** |
| Y-BOCS | Miscellaneous Compulsions | 25. Measures (not checking) to prevent | 0.03 | 0.06 | **0.50** |
| Y-BOCS | Aggressive Obsessions | 41. Fear of doing something else embarrassing | 0.11 | 0.50 | **0.50** |
| Y-BOCS | Contamination Obsessions | 55. Bothered by sticky substances or residues | 0.13 | -0.11 | **0.49** |
| Y-BOCS | Checking Compulsions | 8. Checking that nothing terrible did or will happen | 0.27 | -0.02 | **0.48** |
| Y-BOCS | Contamination Obsessions | 54. Excessive concern with animals | 0.13 | 0.16 | **0.47** |
| Y-BOCS | Aggressive Obsessions | 45. Fear will be responsible for something else terrible happening | 0.05 | 0.15 | **0.47** |
| *Y-BOCS* | *Religious Obsessions* | *60. Excess concern with the right/wrong, morality* | *0.05* | *0.44* | *0.46* |
| Y-BOCS | Sexual Obsessions | 57. Forbidden or perverse sexual thoughts, images, or impulses | -0.15 | 0.22 | **0.46** |
| Y-BOCS | Aggressive Obsessions | 42. Fear will act on unwanted impulses | 0.21 | -0.09 | **0.44** |
| Y-BOCS | Miscellaneous Obsessions | 69. Fear for saying certain things | -0.09 | 0.24 | **0.43** |
| *Y-BOCS* | *Miscellaneous Obsessions* | *70. Fear of not saying just the right thing* | *0.23* | *0.36* | *0.43* |
| Y-BOCS | Aggressive Obsessions | 44. Fear will harm others because not careful enough | -0.01 | 0.31 | **0.42** |
| Y-BOCS | Aggressive Obsessions | 43. Fear steal things | -0.04 | 0.26 | **0.41** |
| Y-BOCS | Miscellaneous Obsessions | 77. Colors with special significance | 0.20 | 0.22 | **0.40** |
| *Y-BOCS* | *Miscellaneous Compulsions* | *22. Need to tell, ask or confess* | *0.10* | *0.32* | *0.40* |
| Y-BOCS | Miscellaneous Obsessions | 78. Superstitions | 0.14 | 0.21 | **0.37** |
| Y-BOCS | Aggressive Obsessions | 39. Violent or horrific images | 0.02 | 0.13 | **0.35** |
| *Y-BOCS* | *Contamination Obsessions* | *51. Concerns or disgust with bodily waste or secretions* | *0.09* | *0.30* | *0.34* |
| *Y-BOCS* | *Religious Obsessions* | *59. Concerned with sacrilege and blasphemy* | *-0.06* | *0.33* | *0.34* |
| Y-BOCS | Cleaning/Washing Compulsions | 5. Actively taking measures to avoid contact with contaminants or other feared objects | -0.08 | -0.01 | **0.20** |

In bold, items with high loadings (>꘡0.20꘡) for one factor but with low loadings (<꘡0.10꘡) for the others. In italics, 11 items with ambiguous classification because of a high loading (>0.2) on two factors or a loading difference <0.1 between the factors (all items were from the Y-BOCS: items 15, 19, 21, 22, 36, 56, 57, 59, 60, 68). RBS-R: Responsiveness Behavioral Scale-Revised; Y-BOCS: Yale-Brown Obsessive Compulsive Scale.

**Supplementary Table 5.** Item loading for the three factors yielded an analysis factor for the items of the Y-BOCS and the RBS-R, and domains related to each item among male participants

| **Scale** | **Domain** | **Item** | **Factor loadings (% of variance)** | | |
| --- | --- | --- | --- | --- | --- |
|  |  |  | **Factor 1 (11.9%)** | **Factor 2 (9.37%)** | **Factor 3 (5.51%)** |
| Y-BOCS | Checking Compulsions | 7. Checking that did not/will not harm self | **0.63** | 0.06 | -0.02 |
| Y-BOCS | Contamination Obsessions | 53. Excessive concern with environmental contaminants | **0.62** | 0.02 | 0.06 |
| Y-BOCS | Aggressive Obsessions | 40. Fear of blurting out obscenities or insults | **0.62** | 0.03 | 0.03 |
| *Y-BOCS* | *Aggressive Obsessions* | *41. Fear of doing something else embarrassing* | *0.60* | *0.01* | *0.17* |
| Y-BOCS | Aggressive Obsessions | 44. Fear will harm others because not careful enough | **0.60** | 0.02 | 0.02 |
| Y-BOCS | Contamination Obsessions | 56. Concerned will get ill because of contaminant | **0.60** | 0.06 | 0.09 |
| Y-BOCS | Aggressive Obsessions | 38. Fear might harm others | **0.60** | -0.02 | 0.03 |
| Y-BOCS | Aggressive Obsessions | 42. Fear will act on unwanted impulses | **0.60** | 0.05 | 0.01 |
| Y-BOCS | Checking Compulsions | 6. Checking that did not/will not harm others | **0.58** | -0.05 | -0.01 |
| Y-BOCS | Checking Compulsions | 8. Checking that nothing terrible did or will happen | **0.57** | 0.06 | 0.04 |
| Y-BOCS | Aggressive Obsessions | 45. Fear will be responsible for something elseterrible happening | **0.57** | 0.03 | 0.07 |
| Y-BOCS | Miscellaneaous Obsessions | 69. Fear for saying certain things | **0.55** | -0.01 | 0.03 |
| *Y-BOCS* | *Miscellaneous Obsessions* | *70. Fear of not saying just the right thing* | *0.54* | *0.04* | *0.17* |
| *Y-BOCS* | *Aggressive Obsessions* | *43. Fear steal things* | *0.53* | *0.01* | *-0.11* |
| *Y-BOCS* | *Contamination Obsessions* | *55. Bothered by sticky substances or residues* | *0.53* | *0.09* | *0.24* |
| *Y-BOCS* | *Religious Obsessions* | *60. Excess concern with the right/wrong, morality* | *0.52* | *0.01* | *0.24* |
| *Y-BOCS* | *Miscellaneous Compulsions* | *22. Need to tell, ask or confess* | *0.52* | *0.03* | *0.278* |
| Y-BOCS | Contamination Obsessions | 54. Excessive concern with animals | **0.51** | 0.06 | 0.01 |
| *Y-BOCS* | *Miscellaneous Compulsions* | *25. Measures (not checking) to prevent* | *0.51* | *0.02* | *0.18* |
| *Y-BOCS* | *Miscellaneous Obsessions* | *77. Colors with special significance* | *0.51* | *0.09* | *0.13* |
| *Y-BOCS* | *Contamination Obsessions* | *52. Concern with dirt or germs* | *0.51* | *0.07* | *0.15* |
| *Y-BOCS* | *Miscellaneous Compulsions* | *20. Mental rituals* | *0.50* | *-0.04* | *0.29* |
| *Y-BOCS* | *Checking Compulsions* | *9. Checking that did not make mistake* | *0.50* | *-0.06* | *0.33* |
| *Y-BOCS* | *Sexual Obsessions* | *57. Forbidden or perverse sexual thoughts, images, or impulses* | *0.50* | *0.02* | *0.28* |
| Y-BOCS | Somatic Obsessions | 79. Concern with illness or disease | **0.49** | 0.03 | 0.04 |
| *Y-BOCS* | *Aggressive Obsessions* | *39. Violent or horrific images* | *0.49* | *0.06* | *0.19* |
| *Y-BOCS* | *Moarding/Saving Obsessions* | *58. Moarding/saving obsessions* | *0.48* | *0.02* | *0.20* |
| *Y-BOCS* | *Miscellaneous Obsessions* | *73. Intrusive images* | *0.47* | *0.08* | *0.12* |
| *Y-BOCS* | *Miscellaneous Obsessions* | *72. Fear of losing things* | *0.45* | *0.09* | *0.25* |
| *Y-BOCS* | *Checking Compulsions* | *11. Checking tied to somatic obsessions* | *0.44* | *-0.07* | *0.26* |
| *Y-BOCS* | *Repeating Rituals* | *12. Re-reading or re-writing* | *0.44* | *0.09* | *0.39* |
| *Y-BOCS* | *Miscellaneous Obsessions* | *75. Bothered by certain sounds/noises* | *0.42* | *0.20* | *0.19* |
| *Y-BOCS* | *Miscellaneous Obsessions* | *68. Need to know or remember* | *0.42* | *0.12* | *0.20* |
| *Y-BOCS* | *Miscellaneous Obsessions* | *74. Intrusive nonsense sounds words, or music* | *0.42* | *0.01* | *0.17* |
| *Y-BOCS* | *Somatic Obsessions* | *80. Excessive concern with body part or aspect of appearance* | *0.40* | *0.01* | *0.26* |
| Y-BOCS | Cleaning/Washing compulsions | 5. Actively taking measures to avoid contact with contaminants or other feared objects | **0.40** | 0.01 | -0.03 |
| *Y-BOCS* | *Contamination Obsessions* | *51. Concerns or disgust with bodily waste or secretions* | *0.39* | *0.17* | *0.09* |
| *Y-BOCS* | *Miscellaneous Compulsions* | *28. Superstitious behaviors* | *0.38* | *0.04* | *0.28* |
| *Y-BOCS* | *Aggressive Obsessions* | *37. Fear might harm self* | *0.37* | *0.05* | *0.22* |
| *Y-BOCS* | *Miscellaneous Obsessions* | *78. Superstitions* | *0.6* | *0.08* | *0.16* |
| *Y-BOCS* | *Cleaning/Washing compulsions* | *1. Excessive or ritualized hygiene* | *0.35* | *0.24* | *0.21* |
| *Y-BOCS* | *Religious Obsessions* | *59. Concerned with sacrilege and blasphemy* | *0.33* | *-0.06* | *0.12* |
| *Y-BOCS* | *Checking Compulsions* | *10. Others* | *0.31* | *-0.01* | *0.31* |
| *Y-BOCS* | *Miscellaneous Obsessions* | *76. Lucky/unlucky numbers* | *0.31* | *0.01* | *0.27* |
| Y-BOCS | Miscellaneous Compulsions | 36. Trichotillomania | **0.24** | 0.01 | 0.06 |
| Y-BOCS | *Hoarding / Collectig Compulsions* | *19. Hoarding/collecting compulsions* | 0.24 | 0.03 | 0.21 |
| RBS-R | Sameness Subscale | 38. Insists on routine | 0.14 | **0.57** | 0.02 |
| *RBS-R* | *Compulsive Subscale* | *16. Completeness* | *-0.02* | *0.57* | *0.13* |
| *RBS-R* | *Sameness Subscale* | *29. Placements of objects* | *0.10* | *0.56* | *0.17* |
| RBS-R | Ritualistic Subsacle | 26. Transportation routine | -0.02 | **0.55** | 0.03 |
| RBS-R | Sameness Subscale | 33. Sits certain place | 0.01 | **0.55** | -0.04 |
| *RBS-R* | *Compulsive Subscale* | *21. Repeating* | *0.02* | *0.55* | *0.14* |
| RBS-R | Ritualistic Subscale | 24. Sleeping/bedtime | 0.04 | **0.53** | -0.06 |
| RBS-R | Stereotypy Subscale | 6. Sensory | -0.04 | **0.52** | 0.03 |
| RBS-R | Sameness Subscale | 34. Appearance/behavior of others | 0.18 | **0.52** | 0.01 |
| RBS-R | Compulsive Subscale | 22. Needs to touch | -0.02 | **0.51** | 0.03 |
| RBS-R | Stereotypy Subscale | 3. Finger movements | -0.01 | **0.50** | 0.07 |
| *RBS-R* | *Sameness Subscale* | *32. Walks certain way* | *-0.05* | *0.50* | *0.13* |
| RBS-R | Restricted Subscale | 42. Preoccupied with part of object | 0.05 | **0.49** | 0.05 |
| *RBS-R* | *Sameness Subscale* | *39. Insists on time* | *0.11* | *0.49* | *0.08* |
| *RBS-R* | *Ritualistic Subsacle* | *25. Self-care routine* | *0.02* | *0.49* | *0.18* |
| RBS-R | Stereotypy Subscale | 2. Head movements | 0.03 | **0.49** | 0.04 |
| RBS-R | Sameness Subscale | 37. Difficult transitions | 0.04 | **0.49** | 0.04 |
| *RBS-R* | *Stereotypy Subscale* | *5. Object usage* | *-0.03* | *0.48* | *0.14* |
| *RBS-R* | *Ritualistic Subscacle* | *23. Eating/mealtime* | *0.03* | *0.48* | *-0.02* |
| *RBS-R* | *Sameness Subscale* | *35. Uses certain door* | *-0.01* | *0.48* | *0.16* |
| RBS-R | Ritualistic Subscale | 27. Play/leisure routine | -0.08 | **0.47** | 0.04 |
| RBS-R | Sameness Subscale | 31. No interruption | 0.01 | **0.46** | 0.02 |
| *RBS-R* | *Restricted Subscale* | *43. Preoccupation with movement* | *-0.06* | *0.45* | *0.14* |
| *RBS-R* | *Compulsive Subscale* | *15. Ordering* | *0.09* | *0.44* | *0.31* |
| *RBS-R* | *Self-injurious Subscale* | *12. Rubs/scratches* | *0.11* | *0.44* | *-0.03* |
| RBS-R | Sameness Subscale | 36. Videotapes | -0.05 | **0.44** | 0.07 |
| *RBS-R* | *Restricted Subscale* | *41. Attached to subject* | *0.12* | *0.43* | *0.02* |
| *RBS-R* | *Compulsive Subscale* | *18. Checking* | *0.12* | *0.43* | *0.14* |
| RBS-R | Self-injurious Subscale | 13. Inserts finger/object | -0.01 | **0.43** | -0.02 |
| *RBS-R* | *Sameness Subscale* | *30. No new places* | *0.08* | *0.42* | *0.03* |
| *RBS-R* | *Stereotypy Subscale* | *1. Body movements* | *-0.08* | *0.41* | *0.11* |
| *RBS-R* | *Compulsive Subscale* | *17. Washing* | *0.22* | *0.40* | *0.22* |
| *RBS-R* | *Compulsive Subscale* | *20. Hoarding* | *0.13* | *0.37* | *0.03* |
| *RBS-R* | *Self-injurious Subscale* | *14. Picks skin* | *0.14* | *0.37* | *-0.17* |
| *RBS-R* | *Stereotypy Subscale* | *4. Locomotion* | *-0.08* | *0.37* | *0.16* |
| RBS-R | Self-injurious Subscale | 11. Pulls hair/skin | 0.03 | **0.36** | -0.04 |
| RBS-R | Ritualistic Subsacle | 28. Communication | 0.08 | **0.36** | 0.01 |
| *RBS-R* | *Self-injurious Subscale* | *7. Hits w/body* | *0.02* | *0.35* | *0.04* |
| *RBS-R* | *Self-injurious Subscale* | *10. Bites self* | *0.18* | *0.35* | *-0.16* |
| RBS-R | Restricted Subscale | 40. Preoccupation with subject | 0.01 | **0.35** | -0.03 |
| RBS-R | Self-injurious Subscale | 8. Hits against surface | 0.07 | **0.33** | -0.06 |
| RBS-R | Self-injurious Subscale | 9. Hits w/object | 0.01 | **0.27** | 0.03 |
| Y-BOCS | Obsessions with need for symmetry or exactness | 64. Obsessions with need for alignment | 0.23 | -0.05 | **0.65** |
| *Y-BOCS* | *Obsessions with need for symmetry or exactness* | *67. Obessions with need to even things out* | *0.23* | *-0.05* | *0.65* |
| *Y-BOCS* | *Ordering/Arranging Compulsions* | *18. Need to make things even or balanced* | *0.24* | *-0.01* | *0.61* |
| *Y-BOCS* | *Obsessions with need for symmetry or exactness* | *62. Obsessions with need for symmetry* | *0.21* | *0.16* | *0.58* |
| *Y-BOCS* | *Compulsions with need of symmetry/exactness* | *17. Compulsions with need of symmetry/exactness* | *0.15* | *0.14* | *0.57* |
| *Y-BOCS* | *Counting Compulsions* | *15. Counting compulsions* | *0.20* | *0.02* | *0.52* |
| *Y-BOCS* | *Ordering/Arranging Compulsions* | *16. Ordering/arranging compulsions* | *0.19* | *0.22* | *0.47* |
| *Y-BOCS* | *Obsessions with need for symmetry or exactness* | *61. Obsessions with need for exactness* | *0.28* | *0.18* | *0.47* |
| *Y-BOCS* | *Miscellaneous Compulsions* | *27. Ritualized eating behaviors* | *0.14* | *0.18* | *0.44* |
| *RBS-R* | *Compulsive Subscale* | *19. Counting* | *0.06* | *0.26* | *0.40* |
| *Y-BOCS* | *Cleaning/Washing compulsions* | *4. Cleaning of household items, inanimate objects or pets.* | *0.29* | *0.05* | *0.40* |
| *Y-BOCS* | *Miscellaneous Compulsions* | *24. Need to touch, tap, or rub* | *0.26* | *0.11* | *0.40* |
| *Y-BOCS* | *Miscellaneous Compulsions* | *21. Rituals involving blinking or staring* | *0.27* | *0.11* | *0.32* |

In bold, items with high loadings (>꘡0.20꘡) for one factor but with low loadings (<꘡0.10꘡) for the others. In italics, 11 items with ambiguous classification because of a high loading (>0.2) on two factors or a loading difference <0.1 between the factors (all items were from the Y-BOCS: items 15, 19, 21, 22, 36, 56, 57, 59, 60, 68). RBS-R: Responsiveness Behavioral Scale-Revised; Y-BOCS: Yale-Brown Obsessive Compulsive Scale

These sub-analyses yielded consistent results. Although the proportion of variance explained by the three FAs representing the same three dimensions varied, the item composition of the three FAs remained consistent with the FAs from Supplementary Table 4 .
