# Supplementary figures and images for: Exploring the multidimensional nature of repetitive and restricted behaviors and interests (RRBI) in autism: neuroanatomical correlates and clinical implications
Source: Mol Autism. 2023 Nov 27;14:45. doi: 10.1186/s13229-023-00576-z (PMC10680239; doi:10.1186/s13229-023-00576-z)

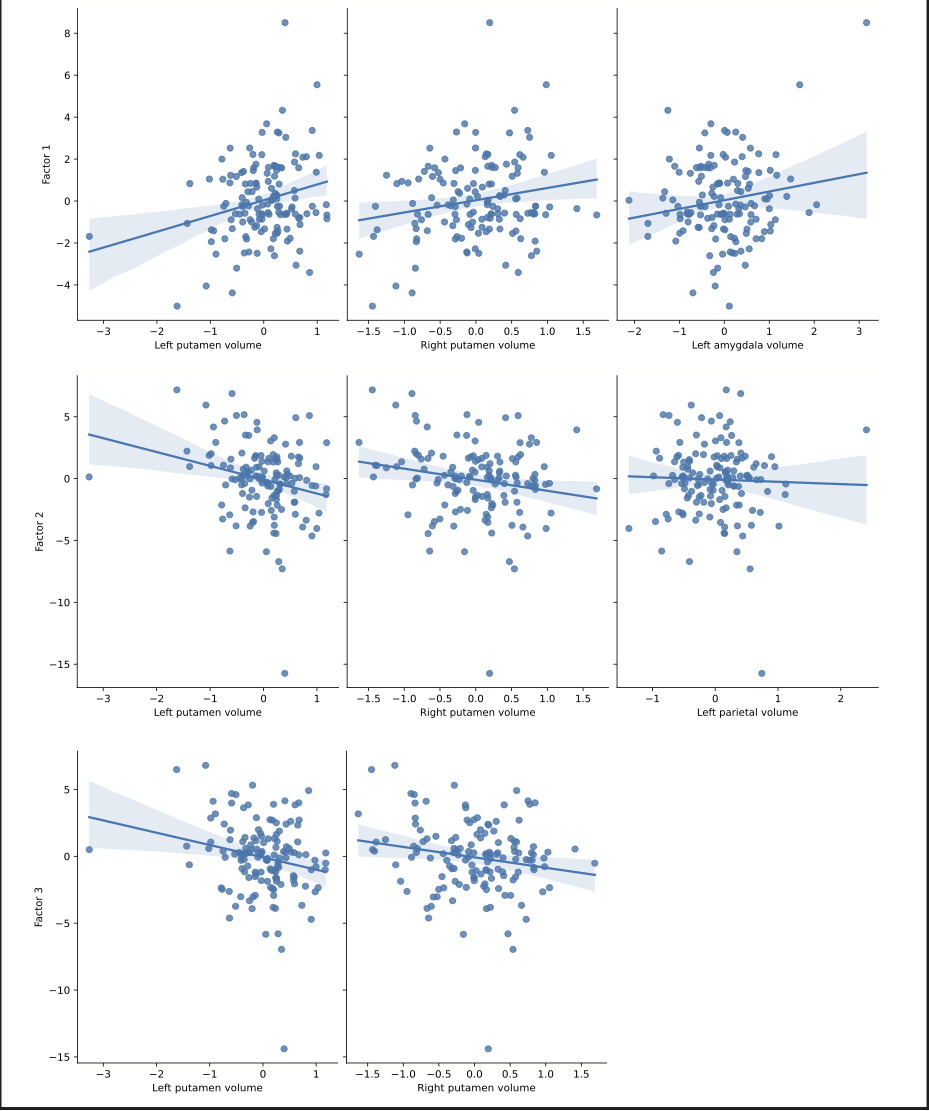

Supplement: Supplementary file 2 — Additional file 2: Fig. S1. Linear regressions between repetitive and restricted behaviors and interests-related factors and the neuroanatomic volumes with a high absolute coefficient and a non-corrected p value<0.05. At the top, the figures represent the regressions between Factor 1 and the left putamen volume (at the left), the right putamen volume (in the middle) and the left amygdala volume (at the right). At the middle, the figures represent the regressions between Factor 2 and the left putamen volume (at the left), the right putamen volume (in the middle) and the left parietal volume (at the right). At the bottom, the figures represent the regressions between Factor 3 and the left putamen volume and the right putamen volume. The figures show an outsider to FA values. Excluding this outsider, the linear regressions between the three FA and the left and right putamen structures maintain a non-corrected p value<0.05, not between Factor 1 and the left amygdala volume, nor between Factor 2 and the left parietal volume. [file 13229_2023_576_MOESM2_ESM.png]

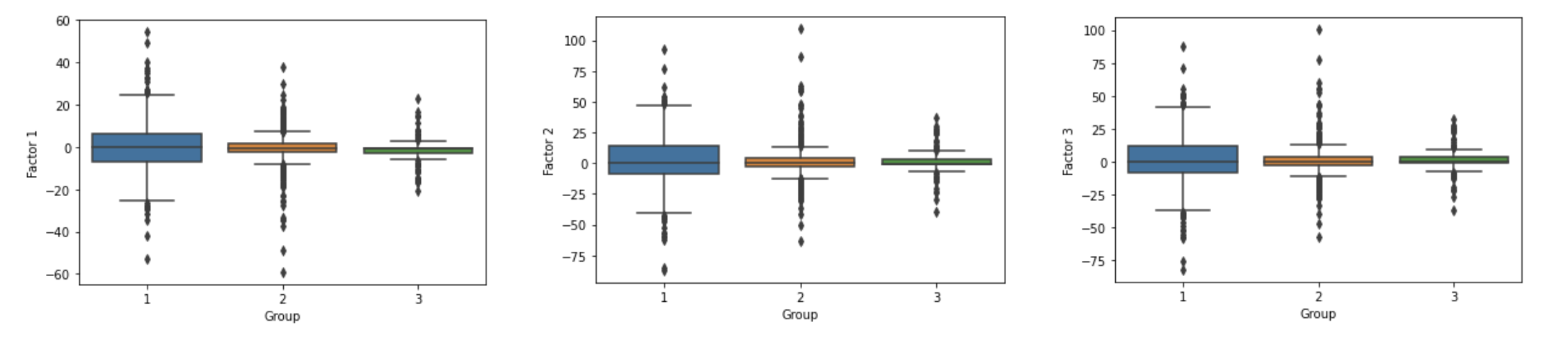

Supplement: Supplementary file 3 — Additional file 3: Fig. S2. Boxplots representing the distribution of individual scores by group [autistic (in blue), relatives (in orange) and controls (in red) groups] for FA1 (at the left), FA2 (in the middle), FA3 (at the right). [file 13229_2023_576_MOESM3_ESM.png]
